# Comparing the Degradation Pathways of Hydrochlorothiazide and Sulfamethoxazole Using Ozone, Anodic Oxidation, and Electro-Fenton Processes

**DOI:** 10.1021/acsestwater.5c00894

**Published:** 2026-05-11

**Authors:** Nadia Gadi, Rebecca Dhawle, Allisson Barros de Souza, Nadine C. Boelee, Deirdre Cabooter, Dionissios Mantzavinos, Raf Dewil

**Affiliations:** † Nijhuis Industries, P.O. Box 44, 7000 AA Doetinchem, The Netherlands; ‡ Department of Chemical Engineering, Process and Environmental Technology Lab, KU Leuven, 2860 Sint-Katelijne-Waver, Belgium; § Department of Chemical Engineering, University of Patras, Caratheodory 1, University Campus, GR-26504 Patras, Greece; ∥ 6426 Agilent Technologies Deutschland, Hewlett-Packard-Strasse 8, 76337 Waldbronn, Germany; ⊥ KU Leuven, Department of Pharmaceutical and Pharmacological Sciences, Pharmaceutical Analysis, Herestraat 49, 3000 Leuven, Belgium; # Department of Engineering Science, University of Oxford, OX1 2JD Oxford, U.K.

**Keywords:** AOPs, eAOPs, wastewater treatment, electrooxidation, micropollutants, emerging contaminants

## Abstract

This study compared the performance of ozonation (O_3_), anodic oxidation (AO), and electro-Fenton (EF) as advanced oxidation processes (AOPs) in the degradation and mineralization of two prevalent pharmaceutical pollutants: sulfamethoxazole (SMX) and hydrochlorothiazide (HCTZ). The effects of varying currents (150–500 mA) on AO and different Fe^2+^ concentrations (0–42 mg L^–1^) on EF were examined. Both EF and O_3_ achieved full removal of SMX and HCTZ, whereas AO resulted in 90% removal. EF demonstrated the highest mineralization efficiency, with 88% total organic carbon (TOC) removal, followed by AO at 74% and O_3_ at 24%. Investigations into the degradation pathways of SMX and HCTZ under each AOP revealed identical degradation mechanisms for EF and AO, with hydroxyl (^•^OH) radicals playing a crucial role. When tested on real municipal effluent, EF showed superior mineralization efficiency and was least affected by the water matrix. This study underscores the effectiveness of EF in the degradation and mineralization of pharmaceutical pollutants, presenting it as a viable option for large-scale wastewater treatment. This work provides the first side-by-side benchmark of O_3_, AO, and the combined AO + EF process for a SMX/HCTZ mixture while jointly evaluating kinetics, TOC mineralization, transformation products, and real-effluent matrix effects.

## Graphical abstract

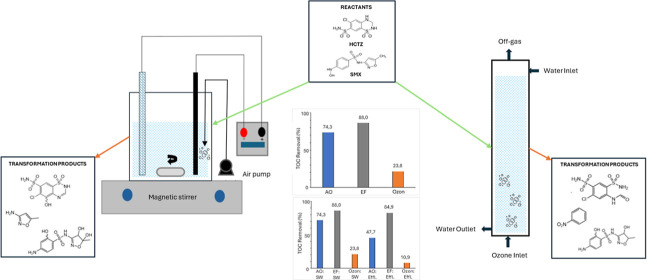

## 1 Introduction

Micropollutants (MPs) in water are a global concern. MPs are represented by several substances, including pesticides, personal care products (PPCPs), pharmaceuticals and industrial compounds. These compounds can enter the environment through wastewater treatment plants (WWTPs) and sewage systems.^1,2^ WWTPs employ a combination of physical, chemical, and biological processes to remove numerous pollutants. However, conventional treatment techniques often result in incomplete removal of MPs, leading to their accumulation in the environment.^3^ The development of analytical techniques has allowed the detection of numerous MPs in surface water and groundwater, despite their low concentrations ranging from a few ng L^–1^ to μg L^–1^.^4^ Even at these low concentrations, MPs can impact aquatic ecosystems and contaminate drinking water sources.^5^ Consequently, with stricter legislation governing water quality standards and advanced analytical screening methods, the scientific community has focused on techniques that can effectively eliminate MPs.

The challenge of removing MPs from water has drawn significant attention to the application of oxidation processes.^6^ Advanced oxidation processes (AOPs) represent a group of water treatment technologies that operate under environmentally relevant conditions. They work by generating highly oxidative hydroxyl radicals (HO^•^) that result in the complete mineralization of organic pollutants.^7^ Owing to their highly reactive nature, the generated HO^•^ interact rapidly with organic compounds present in the matrix, often with rate constants as high as 10^6^ to 10^10^ M^–1^ s^–1^.^8^ This interaction theoretically leads to complete mineralization into water and CO_2_, thus preventing the accumulation of organic micropollutants.^9^ AOPs are not only considered environmentally friendly, but efforts are also being made to make existing AOPs more economically feasible with wide applicability.^10–14^ AOPs can be classified into several categories, such as chemical, electrochemical (eAOPs), sonochemical, photocatalytic, and ozone-based AOPs, which differ in terms of how HO^•^ are generated in situ.^1,15^

Ozonation is a leading technology for the abatement of persistent pollutants on a full scale. Originally used for disinfecting drinking water, it has been applied to municipal wastewater treatment for more than a decade.^16^ Ozonation degrades organic pollutants either directly with ozone (O_3_) or via the HO^•^ formed by O_3_ decomposition in the water matrix.

The attractiveness of O_3_-based AOPs lies in their features, such as no sludge formation, on-site installation, oxygen feed gas use, and quick decomposition of O_3_ to oxygen.^17^ Switzerland has pioneered full-scale ozonation systems in municipal WWTPs for micropollutant treatment, aiming for 80% pollution abatement and several WWTP upgrades by 2040 under the Swiss Water Protection Act.^5^ As EU water legislation becomes stricter, several European countries are starting to use ozonation as a full-scale tertiary treatment technology. However, data comparing the performance of eAOPs with that of standard ozonation are scarce.

eAOPs encompass a variety of processes, including peroxy-coagulation, electro-Fenton (EF), sono electro-Fenton and anodic oxidation (AO). The popularity of these processes has been increasing due to their relative cleanliness, versatility, negligible need for additional chemicals, low energy requirements, and high efficiency toward complete mineralization. eAOPs degrade and mineralize pollutants by producing highly oxidative radicals, either on the surface of the working electrode or by in situ generation of radicals such as hydroxyl radicals (HO^•^), peroxide, and sulfate radicals in the bulk of the solution via chemicals present in the water matrix.^18^ Among these processes, EF and AO have demonstrated substantial potential for removing a wide range of water pollutants.^19^

Beyond pharmaceuticals, electrochemical and hybrid electrochemical AOPs have been increasingly explored for both natural water and industrial wastewater matrices. For example, peroxi-coagulation/electro-Fenton-based treatments have been applied to real textile wastewater, achieving substantial TOC abatements,^20^ and continuous-flow electrocoagulation coupled with (photo)­electro-Fenton has been demonstrated to achieve high mineralization for dye solutions.^21^ Recent reviews further highlight that aerated iron electrocoagulation (and related peroxi-coagulation configurations) can combine separation and oxidation mechanisms and can be adapted for diverse waters and wastewaters.^22^

AO, as described by Lozano et al., can be considered either (i) a heterogeneous oxidation process, where the pollutants are transferred to the surface of the anode from the bulk solution, adsorbed, and undergo an electrochemical electron transfer to produce products that are later desorbed from the anode surface, or (ii) a homogeneous oxidation process that results from the oxidants generated in the bulk solution due to water oxidation or the addition of external ions.^23^ The mechanism for generating HO^•^ on the anode surface is expressed in eq 1. However, this process is constrained by mass transfer of the pollutants from the liquid bulk to the anode and the type of electrode used, since AO relies on oxidation via the HO^•^ formed at the anode surface.^24^

EF, a more potent treatment technology, is an enhanced version of the Fenton process, which uses an Fe^2+^/H_2_O_2_ mixture, or Fenton’s reagent, to produce HO^•^ (eq 2) and degrade organic pollutants (R), oxidizing them until mineralization is achieved (eq 3). This process takes advantage of both direct and indirect oxidation via HO^•^. During EF, hydrogen peroxide (H_2_O_2_) is produced in situ at the cathode via a two-electron reduction of dissolved oxygen (O_2_) (eq 4), thereby eliminating risks associated with the storage and handling of H_2_O_2_. H_2_O_2_ is then activated by the added Fe^2+^ catalyst to form HO^•^ via the well-known Fenton reaction (eq 2).^24^ Furthermore, the Fe^2+^ catalyst, which is added in catalytic amounts, is regenerated electrochemically from the reduction of Fe^3+^ (eq 5), thus ensuring a continuous supply of HO^•^ via the Fenton reaction (eq 2) to the solution being treated^19^ and avoiding the formation of large quantities of iron sludge.^25^ However, EF has significant limitations. Its efficiency is highly dependent on pH, with a peak at pH 3. If the pH is increased to 4, Fe^2+^ precipitates in the form of iron hydroxide, and Fe^3+^ forms complexes that hinder the removal of the target compound, making the process ineffective for removing pollutants.^25^

Therefore, the choice of electrodes is of utmost importance in both EF and AO processes. Carbonaceous cathodes are often used for these processes because of their high hydrogen evolution potential and stability, enabling them to efficiently generate Fenton’s reagents.^26^ Nonactive anodes such as Boron Doped Diamond (BDD), PbO_2,_ and IrO_2_ display higher performance than do active anodes such as platinum because of the larger amount of M­(HO^•^) radicals generated on their surface from the water oxidation reaction (eq 1).^2^ Nonactive anodes have oxygen evolution potentials ranging from 1.2 to 2.6 V versus SHE, which are much larger than those of active anodes. A specific feature of nonactive anodes is their ability to generate HO^•^ through water discharge. These radicals are physisorbed on the electrode surface, making them more available for reactions than those formed on active anode materials such as platinum or titanium oxide.^9^ BDD anodes are particularly popular because of their high O_2_ evolution potential (2.3 V vs SHE), enabling complete mineralization of the target pollutant. BDD is also characterized by a low corrosion rate, low background current, high electrochemical stability, wide potential window, and ease of operation and surface cleaning, making it an excellent choice for electrochemical processes. Furthermore, in the presence of sulfate ions, BDD electro-generates sulfate radicals SO_4_^•–^ next to HO^•^ (eqs 6 and 7), improving the overall removal of organics from the system.^27^ BDD electrodes allow greater contact between pollutants and electrogenerated HO^•^ radicals due to the weak adsorption of these radicals on their surface. Owing to its long service life, inertness toward pollutants, and high activity, BDD is one of the most used anodes against which most of the new modified electrodes are compared.^28^

1 $$M+H_{2}O\to M({HO}^{\cdot })+H^{+}+e^{-}$$

2 $${Fe}^{2+}+H_{2}O_{2}\to {Fe}^{3+}+{HO}^{\cdot }+{OH}^{-}$$

3 $$M({HO}^{\cdot })+R\to M+{mCO}_{2}+{nH}_{2}O+H^{+}+e^{-}$$

4 $$O_{2}+{2H}^{+}+2e^{-}\to H_{2}O_{2}$$

5 $${Fe}^{3+}+e^{-}\to {Fe}^{2+}$$

6 $$HS{O_{4}}^{-}\to S_{2}{O_{8}}^{2-}+{2H}^{+}+{2e}^{-}$$

7 $$BDD+S{O_{4}}^{2-}\to BDD\left(S{O_{4}}^{\cdot -}\right)+e^{-}$$

Although ozonation and electrochemical advanced oxidation processes (eAOPs) have been studied extensively, direct, side-by-side comparisons for pharmaceutical mixtures that simultaneously address degradation kinetics, mineralization, and transformation product formation (especially in real effluents) remain scarce. The goal of this work is to fill this gap by benchmarking ozonation (O_3_), anodic oxidation (AO), and the combined AO + electro-Fenton process (EF + AO) for the simultaneous degradation of sulfamethoxazole (SMX) and hydrochlorothiazide (HCTZ). We quantify removal kinetics and TOC mineralization, elucidate transformation products and possible degradation pathways for each process, and assess matrix effects in real municipal effluent. A preliminary energy and operating-cost comparison is also provided to support practical implementation.

## 2 Materials and Methods

### 2.1 Chemicals

Hydrochlorothiazide (99%) and sulfamethoxazole (98%) were procured from Sigma-Aldrich (Zwijndrecht, Netherlands) and Thermo Scientific Chemicals (Breda, Netherlands), respectively, and were used as target contaminants. Ferrous sulfate (FeSO_4_·7H_2_O, 99%; Acros Organics, Geel, Belgium) was used as the Fe^2+^ source to activate H_2_O_2_ during the Fenton reaction. Sodium sulfate (Na_2_SO_4_; Boom Chemicals, Meppel, Netherlands) was used as a supporting electrolyte at 50 mM (7.1 g L^–1^) to increase solution conductivity. Sulfuric acid (96%; Thermo Scientific, Breda, Netherlands) was used for pH adjustment. Acetonitrile (CH_3_CN, 99.9%) and phosphoric acid (H_3_PO_4_, 85%) were obtained from Sigma-Aldrich (Schnelldorf, Germany) and used for HPLC and LC–MS analyses. Ultrapure water was produced with a Milli-Q system (18 MΩ cm resistivity; Merck, Darmstadt, Germany). Simulated wastewater was prepared by dissolving SMX and HCTZ (40 mg L^–1^ each) in demineralized water (0.1–5 μS cm^–1^). For the AO and EF + AO experiments, Na_2_SO_4_ was added to reach 50 mM. The composition of the real municipal effluent is listed in Table 1.

**Table 1:** Composition of the Real Effluent Used in the Experiments

component	concentration (mg L^–1^)
DO	11.5
COD	53.2
NH_4_^+^	0.8
NO_3_^–^	3.8
NO_2_^–^	0.67
PO_4_^–^	0.21
Cl^–^	236.7
SO_4_^2–^	9.1
Ca^2+^	98
HCO_3_^–^	214

### 2.2 Setup

#### 2.2.1 Anodic Oxidation and Electro-Fenton Processes

Electrochemical experiments were performed in an undivided cell with a working volume of 1 L. The cell consisted of a cylindrical compartment (10 cm diameter, 15 cm height) housing a BDD mesh anode (5 cm × 10 cm, NeoCoat, Switzerland) facing a carbon felt (CF) cathode (5 cm × 10 cm; Mersen BV Benelux), with an interelectrode gap of 1 cm. Air was introduced through a fritted glass tube to ensure sufficient dissolved oxygen for in situ H_2_O_2_ production at the cathode. To increase conductivity, Na_2_SO_4_ was added to reach 50 mM (7.1 g L^–1^). A power supply (RIGOL DP711, United Kingdom) provided constant current, and the solution was continuously mixed with magnetic stirring to maintain homogeneity. For EF + AO experiments, the pH was adjusted to 3.0 ± 0.1 using H_2_SO_4_ and Fe^2+^ was added prior to treatment. AO experiments were carried out without added Fe^2+^ and without pH adjustment (native pH of the matrix, 7–8) unless stated otherwise.

The electrochemical operating conditions were selected to provide a reproducible benchmark and to align with commonly reported BDD/CF systems. The 1 cm interelectrode distance was fixed to limit ohmic losses while still allowing efficient mixing and gas dispersion. The tested currents (150–500 mA) correspond to current densities of 3–10 mA cm^–2^ for the 50 cm^2^ electrodes, which is within the range typically applied in BDD-based anodic oxidation and electro-Fenton studies.^25,26^ The Na_2_SO_4_ supporting electrolyte (50 mM) ensured sufficient conductivity and promoted the formation of reactive oxygen species during BDD electrolysis.^18,27^

#### 2.2.2 Ozone

Ozonation experiments were carried out in a pilot-scale reactor with a working volume of 10 L operating in batch mode. The synthetic matrix and the real municipal effluent were treated at their native pH (7–8) without adjustment. For comparison, EF + AO experiments were performed at pH 3.0 ± 0.1 (Section 2.2.1). O_3_ was generated by corona discharge using oxygen as the feed gas, which was subsequently purged through the reaction mixture prior to commencing the experiments. The required concentration of O_3_ was determined based on the initial total organic carbon (TOC) content of the reaction mixture. For each experiment, O_3_ dosing was capped at a maximum of 2 g O_3_ g^–1^ TOC. The reaction time required to achieve the maximum O_3_ dose at the calculated O_3_ concentration was determined theoretically, and this time was used to define the end point of the experiments. The concentration of ozone in the inlet and outlet gas was measured throughout the experiment via BMT meters. The real ozone concentration entering the system at any given time was then corrected using the inlet and outlet ozone concentrations and the gas flow rate such that the average was 1.8 g O_3_ h^–1^. Samples were extracted at various O_3_ dose levels via a sampling valve integrated into the reactor. An online monitoring system (real-time TOC via a Sievers InnovOx system) was connected to the reactor, enabling real-time tracking of the ozonation process.

## 3 Analytical Methods

### 3.1 Degradation Kinetics

The concentrations of SMX and HCTZ were analyzed via an Agilent 1260 Infinity II Prime HPLC system (Agilent Technologies, Waldbronn, Germany). The system consisted of a binary pump, an autosampler, a column compartment and a diode array detector (DAD). Chromatographic separations were carried out on a Poroshell 120 SB-Aq column (100 × 3.0 mm; particle size (*d*_p_) = 2.7 μm; Agilent Technologies) using a mobile phase consisting of water (A) and acetonitrile (B), both acidified with 0.1% orthophosphoric acid, in gradient elution mode. The gradient elution method started at 5% B for 0.5 min, then increased from 5% to 50% B in 7.5 min, followed by a gradient increase to 100% B in 1 min, held for 2 min. B was reset to initial conditions (5% B) in 0.1 min and held for 2 min for column re-equilibration. The injection volume, mobile phase flow rate, column temperature and UV absorbance wavelength were 5 μL, 0.80 mL min^–1^, 40 °C and 270 nm, respectively. Under these conditions, SMX and HCTZ eluted at 4.35 and 2.65 min, respectively. A linear calibration curve, including 7 concentrations between 1 and 50 mg·L^–1^ SMX and HCTZ, was constructed (*n* = 3, *R* ^2^ > 0.997) to determine the concentrations of SMX and HCTZ.

### 3.2 Mineralization Assessment

To track the removal of organic matter during the treatment, samples were collected at predetermined intervals. The total organic carbon (TOC) content was measured via a Shimadzu TOC analyzer (TOC-LCSH/CSN Standalone, Shimadzu,’s-Hertogenbosch, Netherlands). TOC removal was calculated via eq 8

8 $$TOC\;removal\;\%=\frac{{TOC}_{0}-{TOC}_{t}}{{TOC}_{0}}\times 100$$

with TOC_0_ (mg·L^–1^) the value before treatment and TOC_*t*_ (mg·L^–1^) the value at time *t*.

The mineralization current efficiency was calculated as follows

9 $$MCE\;\left(\%\right)=\frac{n\times F\times V\times \Delta TOC}{4.32\times 10^{7}\times m\times I\times t}\times 100$$

where *n* is the number of electrons responsible for the mineralization reaction, *V* is the total volume of the reacting mixture (L), *t* is the reaction time (h), *m* is the number of carbon atoms in the reactants, *I* is the applied current (A), *F* is Faraday’s constant (96487 C mol^–1^), and ΔTOC is the reduction in the TOC from the initial value to a given time. The number of electrons and the number of carbon atoms in the reactants were taken from the mineralization reactions of SMX and HCTZ shown below.

10 $$C_{10}H_{11}N_{3}O_{3}S+21H_{2}O\to {10CO}_{2}+41H^{+}+S{O_{4}}^{2-}+3N{H_{4}}^{+}+{42e}^{-}$$

11 $$C_{7}H_{8}{ClN}_{3}O_{4}S_{2}+24H_{2}O\to {7CO}_{2}+52H^{+}+2S{O_{4}}^{2-}+N{H_{4}}^{+}+2N{O_{3}}^{-}+{Cl}^{-}+{46e}^{-}$$

### 3.3 Identification of Transformation Products

An Agilent 1290 Infinity II UHPLC system (Agilent Technologies) equipped with a binary pump, a vial sampler and a column compartment coupled to an Agilent 6550 iFunnel quadrupole time-of-flight mass spectrometer (Q-TOF) was utilized to elucidate the transformation products. Chromatographic separation was conducted in gradient elution mode on a reversed-phase column (Zorbax RRHD SB-Aq, 2.1 × 100 mm; *d*_p_ = 1.8 μm; Agilent Technologies) with a mobile phase consisting of water acidified with 0.1% formic acid (FA) (A) and acetonitrile with 0.1% FA (B). The gradient elution method was as follows: 5–45% B in 8 min; 45–95% B in 4 min; 95% B held for 3 min and returned to initial conditions (5% B) in 0.1 min; and 5% B maintained for 3 min for column re-equilibration. The injection volume, column temperature and mobile phase flow rate were set at 5 μL, 40 °C and 0.30 mL min^–1^, respectively. The Q-TOF mass spectrometer was operated in 2 GHz extended dynamic range mode, with ionization performed in both positive and negative electrospray ionization modes via a Dual Agilent Jet Stream Technology Ion Source. The gas temperature, drying gas flow and nebulizer pressure were set at 230 °C, 15 L min^–1^ and 35 ψ, respectively, with a sheath gas temperature of 350 °C and a sheath gas flow of 12 L min^–1^. For positive electrospray ionization, the capillary voltage was set at 3500 V, the nozzle voltage was 0 V, and the fragmentor voltage was 335 V, whereas the operating parameters for negative electrospray ionization mode were −3500 V, 1000 and 335 V, respectively. MS and MS/MS spectra were collected at 3 spectra s^–1^, and MS/MS fragmentation runs were performed at 10, 20, and 40 eV. The chromatograms were processed with Agilent MassHunter Qualitative software (version 10.0), and molecular features were extracted via Agilent MassHunter Profinder (version 10.0.2) and Agilent MassHunter Profiler Professional (version 15.1).

## 4 Results and Discussion

### 4.1 Effect of the Current on the Removal of SMX and HCTZ (AO)

A significant factor in electrochemical AOPs is the applied current, which influences both the quantity and type of oxidative species generated. Thus, the impact of current density on the removal of 40 mg·L^–1^ SMX and 40 mg·L^–1^ HCTZ in 0.05 M Na_2_SO_4_ was studied at 150 mA, 300 mA, and 500 mA, and the results are presented in Figure 1.

**Figure 1:**
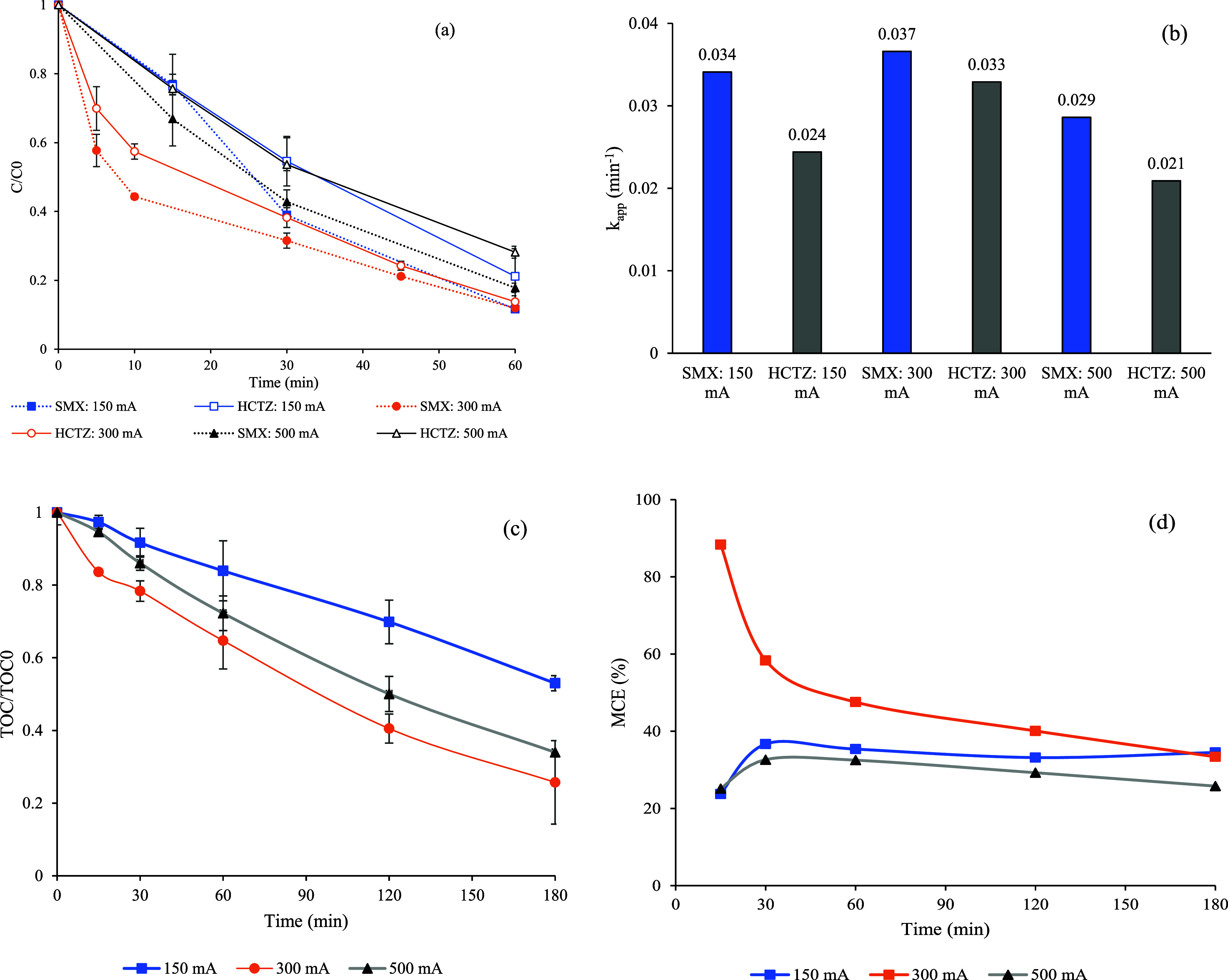
Effect of applied current on the anodic oxidation of [SMX] = 40 mg·L^–1^ and [HCTZ] = 40 mg·L^–1^. (a) Degradation of SMX and HCTZ via anodic oxidation at 150 mA, 300 mA and 500 mA current intensities. (b) The corresponding *k*_app_ (c) mineralization and (d) mineralization current efficiency at 150 mA, 300 mA and 500 mA current intensities.

When the current was increased from 150 mA to 300 mA (Figure 1a), SMX degradation remained stable, whereas HCTZ degradation increased from 78.9% to 86.2%. Mineralization nearly doubled from 16.1% at 150 mA to 35.3% at 300 mA within 60 min (Figure 1c). At 180 min, the TOC removal was 47% and 74.3% at 150 mA and 300 mA, respectively. This enhanced degradation and mineralization with increased current can be attributed to the substantial increase in oxidant species production on the BDD surface (eq 1) and the increased electron transport rate from the pollutant to the BDD surface via direct oxidation. Similar effects of the applied current on the removal of the pharmaceutical compound anastrozole have been reported, with an increase in the applied current from 100 mA to 200 mA, increasing the degradation from 82.4% to 97.5%.^29^

A further increase in the applied current from 300 mA to 500 mA had a negative influence on both SMX and HCTZ degradation and mineralization. The 60 min SMX degradation decreased from 88.1% to 82.1%, whereas the HCTZ degradation decreased from 86.2% to 78.1%. The mineralization also decreased, from 35.3% to 27.7% over 60 min and from 74.3% to 66% over 180 min. A higher applied current may be unfavorable for organic pollutant abatement because HO^•^ generated on the BDD surface are consumed by side reactions producing weaker oxidizing species and parasitic oxygen evolution reactions, leading to lower degradation and mineralization efficiencies.^30^ The persulfate generated from the sulfate ions present in the electrolyte on the surface of the BDD anode (eq 7) and the hydrogen peroxide and hydroperoxyl radicals produced from the generated HO^•^ (eqs 3 and 4) have a lower oxidative power than the HO^•^ radicals and result in poor removal efficiencies of the target pollutant.^30,31^ The lower TOC removal at higher applied currents indicates that the mass transfer of the intermediates is a limiting factor in the AO on the BDD surface.^31^ Tasca et al. reported similar results for the removal of insecticides, where increasing the applied current from 100 mA to 500 mA decreased mineralization from 89.3% to 87.5%.^32^

The apparent rate constant (*k*_app_) for the degradation of both SMX and HCTZ was computed via pseudo-first order kinetics. It was assumed that the degradation of both SMX and HCTZ was dominated by oxidation via HO^•^ generated on the BDD. The *k*_app_ was calculated by plotting the logarithmic decay of the pollutant against time, and the computed *k*_app_ values are shown in Figure 1b along with the corresponding *R* ^2^ values in Table 2. Regardless of the applied current, the *k*_app_ for SMX degradation was greater than that for HCTZ degradation, indicating that the reactivity of SMX with HO^•^ is greater than that of HCTZ. Increasing the current to 500 mA decreased the current efficiency. The highest current efficiency is observed at 300 mA, and at prolonged reaction times, a large share of the applied current is consumed toward parasitic reactions.

**Table 2:** *R* ^2^ Values for the Kinetics of SMX and HCT Degradation at Different Currents

current (mA)	150	300	500
*R* ^2^ HCTZ	0.98	0.89	0.98
*R* ^2^ SMX	0.98	0.96	0.99

### 4.2 Effect of Catalyst Concentration

Fe^2+^ serves as a catalyst during the EF + AO process. Its concentration is crucial because it activates electrogenerated H_2_O_2_ to produce HO^•^ via the Fenton reaction.^33^ To isolate the contribution of the Fenton reaction in the EF + AO configuration, experiments were conducted at fixed pH 3.0 with initial Fe^2+^ concentrations of 0, 11.1, 21.0, and 42.0 mg L^–1^. The condition at 0 mg L^–1^ Fe^2+^ corresponds to AO operated under the same conditions. SMX degradation rates were faster than those of HCTZ for all iron concentrations, which can be explained by the higher aqueous solubility of SMX and the resulting enhanced mass transfer and interaction with oxidative agents.^34^ The results in Figure 2 highlight the importance of adding Fe^2+^: increasing the catalyst concentration from 0 to 11.1 mg L^–1^ approximately doubled the removal rates of both HCTZ and SMX. Apparent pseudo-first-order rate constants (*k*_app_) were calculated and are shown in Figure 2c.

**Figure 2:**
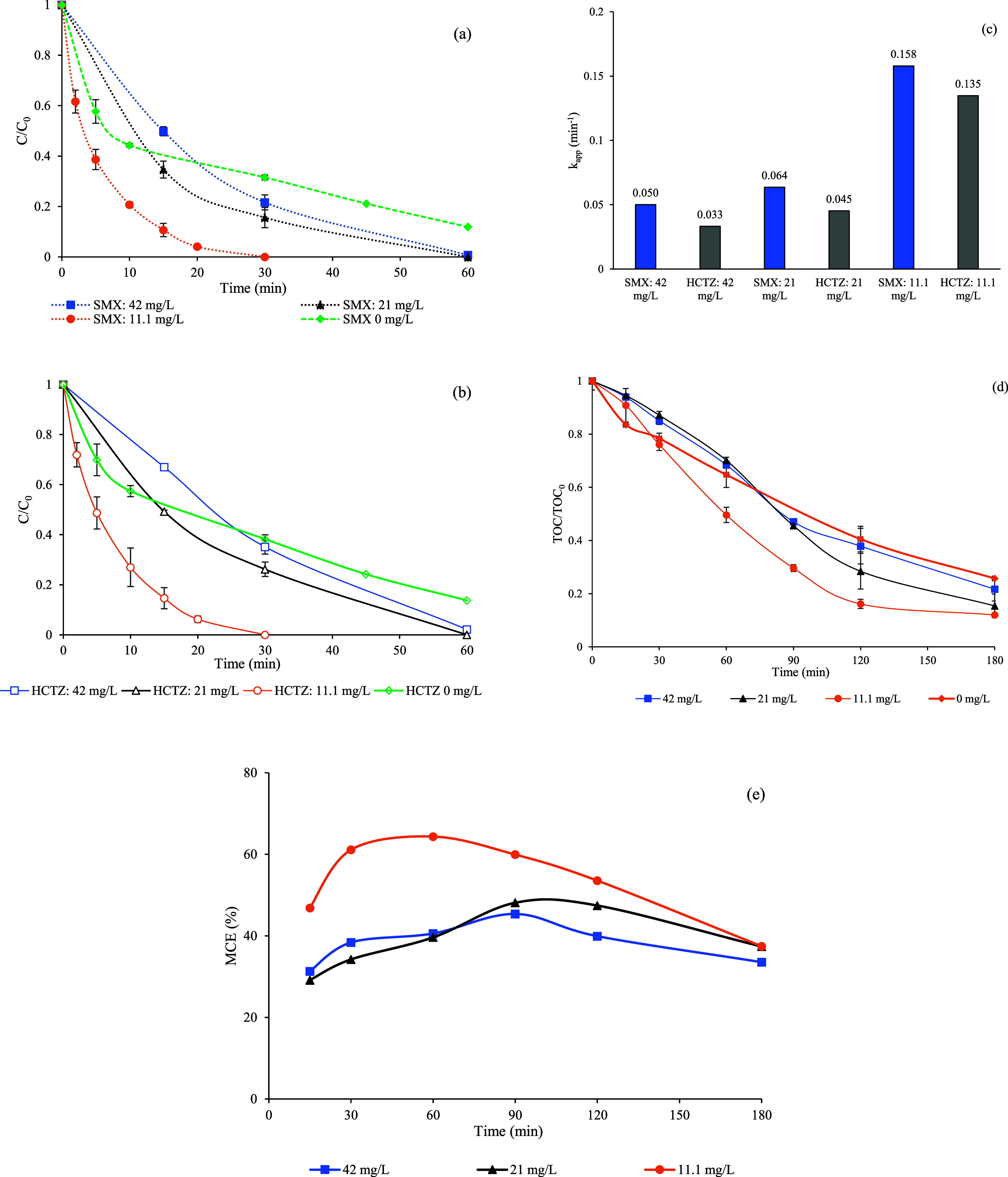
Effect of the initial Fe^2+^ concentration (0, 11.1, 21.0, and 42.0 mg L^–1^) on the removal of [SMX]­0 = 40 mg L^–1^ and [HCTZ]­0 = 40 mg L^–1^ during the combined electro-Fenton and anodic oxidation process (EF + AO). (a) HCTZ degradation, (b) SMX degradation, (c) apparent pseudo-first-order rate constants (*k*_app_), (d) TOC removal, and (e) mineralization current efficiency. Note: Fe^2+^ = 0 mg L^–1^ corresponds to anodic oxidation operated under the same conditions (pH 3.0) without added catalyst.

A similar trend was observed for mineralization. AO (0 mg L^–1^ Fe^2+^) achieved 74% TOC removal after 3 h, whereas EF + AO reached 87% TOC removal at 11.1 mg L^–1^ Fe^2+^ for the same duration. Further increasing Fe^2+^ led to lower *k*_app_ values and slightly lower mineralization, likely due to scavenging of HO^•^ and other side reactions at higher iron levels. After 3 h of EF + AO treatment with 42.0 mg L^–1^ Fe^2+^, TOC removal was only 4% higher than that obtained with AO. This trend is also reflected by the mineralization current efficiency (MCE): as shown in Figure 2e, MCE peaked at about 60% for 11.1 mg L^–1^, while higher concentrations (21.0 and 42.0 mg L^–1^) resulted in maximum MCE values of approximately 45–50%.

### 4.3 Comparison of Removal Efficiencies Using EF + AO, AO and Ozone

Before investigating the degradation pathways of SMX and HCTZ via AO, EF + AO and O_3_ treatments, a comparison of their degradation and mineralization efficiencies was performed, and the results are presented in Figure 3. Ozonation and AO were performed at the native pH of the matrices (7–8), whereas EF + AO experiments were conducted at pH 3.0 ± 0.1. Efficiency was evaluated based on the decay of SMX, HCTZ and TOC over time. The experimental data revealed a consistent trend across the three technologies, with SMX degrading faster than HCTZ. The complete removal of both compounds was achieved after 13 and 18 min for SMX and HCTZ, respectively, when O_3_ was used, and after 30 min for both compounds with EF + AO. The initial stages (after 7 min for SMX and 15 min for HCTZ) of the EF + AO treatment resulted in the fastest degradation rates, mainly due to the higher oxidative potential of HO^•^ (*E*° = 2.8 V) than that of O_3_ (*E*° = 2.08 V).^35^ Following this phase, ozonation exhibited superior efficiency, resulting in faster removal of residual SMX and HCTZ. This can be explained by the decline in SMX and HCTZ concentrations over time due to oxidation and the nonselective nature of HO^•^, which contributes to byproduct degradation. Thus, fewer HO^•^ per molecule of SMX and HCTZ remain.^36^ In contrast, O_3_ molecules, which are more selective, can diffuse and react with targeted compounds quickly, resulting in efficient degradation.^37^ Furthermore, while HO^•^ is a stronger oxidant, it has a shorter lifetime (10^–7^ to 10^–8^ s) than O_3_ (1 min −2 min at neutral pH).^38^ However, the lifetime of HO^•^ in the O_3_–HO^•^ mixture extends up to ∼0.12 s via the O_3_/HO^•^ synergy,^38^ potentially resulting in a higher oxidant-to-pollutant ratio during ozonation. The slower degradation and mineralization rates observed during AO oxidation are likely due to mass transfer limitations. The compounds are predominantly oxidized at the anode surface by the physisorbed M­(HO^•^) during this process, resulting in poor utilization of the formed HO^•^ when the organic matter is not near the anode surface. A different trend was observed for mineralization. The performance of the processes can be classified as EF + AO > AO > O_3_, with TOC removal efficiencies of 88%, 74% and 24%, respectively. The highest mineralization efficiency is achieved by eAOPs, which are HO^•^-based oxidations. These nonselective radicals can react with both the targeted compounds and their degradation intermediates, leading to minimal to no secondary pollution.^15,39^ In contrast, the ozonation process primarily degrades compounds through direct O_3_ molecule reactions, breaking them into smaller fragments, which can sometimes be chemically stable and more persistent in water. Hence, further and prolonged treatments might be required for complete mineralization.^40^

**Figure 3:**
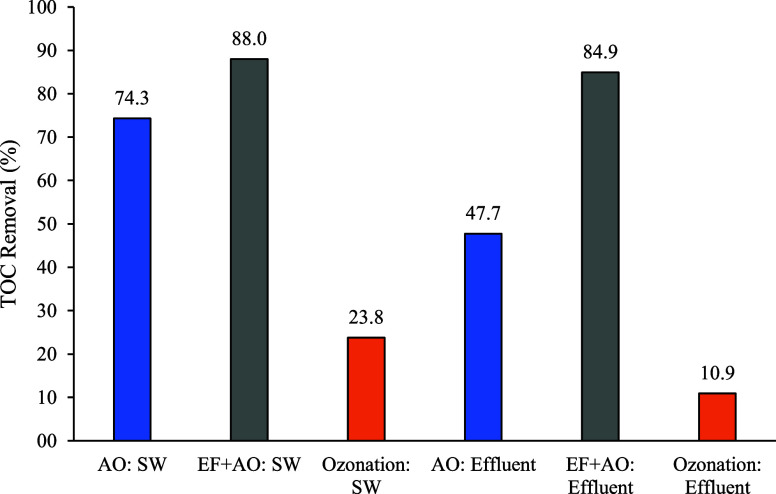
Mineralization in simulated wastewater and real effluent via anodic oxidation, electro-Fenton, and ozonation.

### 4.4 Effect of the Water Matrix on the Removal Efficiency

The role of the water matrix in the mineralization efficiency is displayed in Figure 3. As shown in the figure, the water matrix significantly impacts the total TOC removed during the treatment process. When shifting from simulated water to real effluent, the most substantial decrease in mineralization was observed for ozonation (from 23.76% to 10.9%), followed by AO (from 74.29% to 47.74%). However, the mineralization efficiency of the EF + AO process only slightly decreased (87.9% to 84.9%). Real effluent, which contains a higher concentration of inorganic matter, effluent organic matter (EfOM) and trace amounts of organic contaminants (TrOCs), changes the mineralization efficiency.

The water matrix composition significantly affects AO. The organic and inorganic loads are higher in real effluent, which alters the electrochemical window of the system and thus affects the surface layer reactions occurring at the anode.^41^ In AO, the predominant oxidative species are HO^•^ generated on the surface of the BDD. Changes in surface layer reactions may decrease the production of these HO^•^, leading to lower mineralization. The presence of anionic species such as nitrates (NO_3_^–^), phosphates (HPO_4_^2–^) and bicarbonates (HCO_3_^–^) in real effluent could impede overall TOC abatement, as these inorganic species can scavenge HO^•^, thereby hindering organic removal during treatment.^42^ Chlorides in the water matrix can generate organochloride species that are resistant to mineralization.^43^ Natural organic matter present in real water matrices can block the active sites of the BDD anode, leading to lower mineralization rates.^28^

O_3_, a rather selective oxidant with a high affinity for electron-rich functional groups, is contrasted by the indiscriminate tendency of HO^•^ to oxidize organic pollutants.^44^ Ozonation reactions are influenced by the presence of carbonates (CO_3_^2–^), bicarbonates (HCO_3_^–^), nitrites (NO_2_^–^), and EfOM, which are the primary scavengers of O_3_ and HO^•^ generated during ozonation.^45^ The EfOM present in the effluent has significantly greater reaction rates (*k*_EfOM_ = 1 × 10^3^ M^–1^ s^–1^) with ozone and therefore consumes all the ozone within the system, thus decreasing mineralization.^46^ Moreover, the presence of humic acid in the effluent inhibits the formation of HO^•^, thus reducing indirect oxidation during ozonation. A significant portion of the competition for the oxidizing species comes from CO_3_^2–^ and HCO_3_, which are highly reactive (*k*_O_3__ ≈ 10^6^ to 10^8^ M^–1^ s^–1^) toward O_3_, making O_3_ less available for organic matter mineralization.^47^ During secondary effluent treatment, chlorides may replace electron-rich functional groups such as amines and phenols with chlorinated substitutes, resulting in lower reactivity toward O_3_ and reducing the mineralization efficiency of the ozonation process.^48^ The lower mineralization efficiency with real effluent could also be a result of highly stable molecules (typically EfOM) and the formation of low molecular weight organic compounds from large reactive molecules that are resistant to further oxidation via ozonation.^49^

The least significant effect of the water matrix was noted during the EF + AO process, with only a 3% decrease when shifting from simulated wastewater to real effluent. EF + AO outperforms both AO and ozonation in terms of removal and mineralization in simulated wastewater. Similar results are observed with real effluent, with EF + AO showing nearly 1.5- and 10-fold better removal efficiencies than AO and ozonation, respectively. The superior performance of EF + AO can be attributed to the homogeneous generation of HO^•^ in the bulk solution, unlike the heterogeneous generation of HO^•^ near the anode in AO, thus ensuring better mass transport during EF + AO reactions.^50^

The mineralization efficiency, regardless of the water matrix used, followed the trend of EF + AO > AO > ozonation. Greater mineralization is obtained with simulated wastewater than with real effluent. The presence of several inorganic ions in real effluent with a higher affinity for oxidizing species reduces the TOC removal, particularly in the case of O_3_. The formation of carboxylic acids from EfOM with high resistance to oxidation by HO^•^ in the cases of AO and EF + AO and the highly selective nature of O_3_ could be responsible for the lower mineralization efficiency in real effluent.^19,50^

## 5 Identification of SMX and HCTZ Transformation Products

### 5.1 Sulfamethoxazole

The transformation products (TPs) produced during treatment with advanced oxidation processes were tentatively identified by UHPLC-QTOF-MS in simulated wastewater. Their elemental formula, exact mass, accurate *m*/*z*, retention time and mass accuracy are reported in the Supporting Information in Table S1. Figure 4a depicts the potential degradation pathway for the electrochemical removal of SMX by AO and EF + AO. Owing to the generation of highly reactive and unselective HO^•^, the primary transformation mechanism involves an increase in the O/C ratio. This signifies an oxygen transfer mechanism via hydroxylation of the aromatic ring, resulting in the formation of SMX-269, SMX-285 and SMX-301, which correspond to mono-, di- and trihydroxylated intermediates, respectively. Additionally, the isoxazole ring was susceptible to HO^•^ attack, leading to the formation of SMX-287 and SMX-303. These pathways align with previous literature, where HO^•^ was identified as the main oxidant, leading to the generation of hydroxylated SMX byproducts.^51,52^ Another transformation pathway involves deamination of the aromatic ring, followed by HO^•^ attack, resulting in the formation of SMX-254 (mono–OH), SMX-270 (di–OH) and SMX-286 (tri–OH).^53^ The generation of SMX-98 (3-amino-5-methylisoxazole) is a result of sulfonamide cleavage by HO^•^ attack, whereas C–S bond dissociation and the loss of an aniline moiety led to the formation of a hydroxylamine intermediate (SMX-173), which is then oxidized to form its nitroso-derivative (SMX-171).

**Figure 4:**
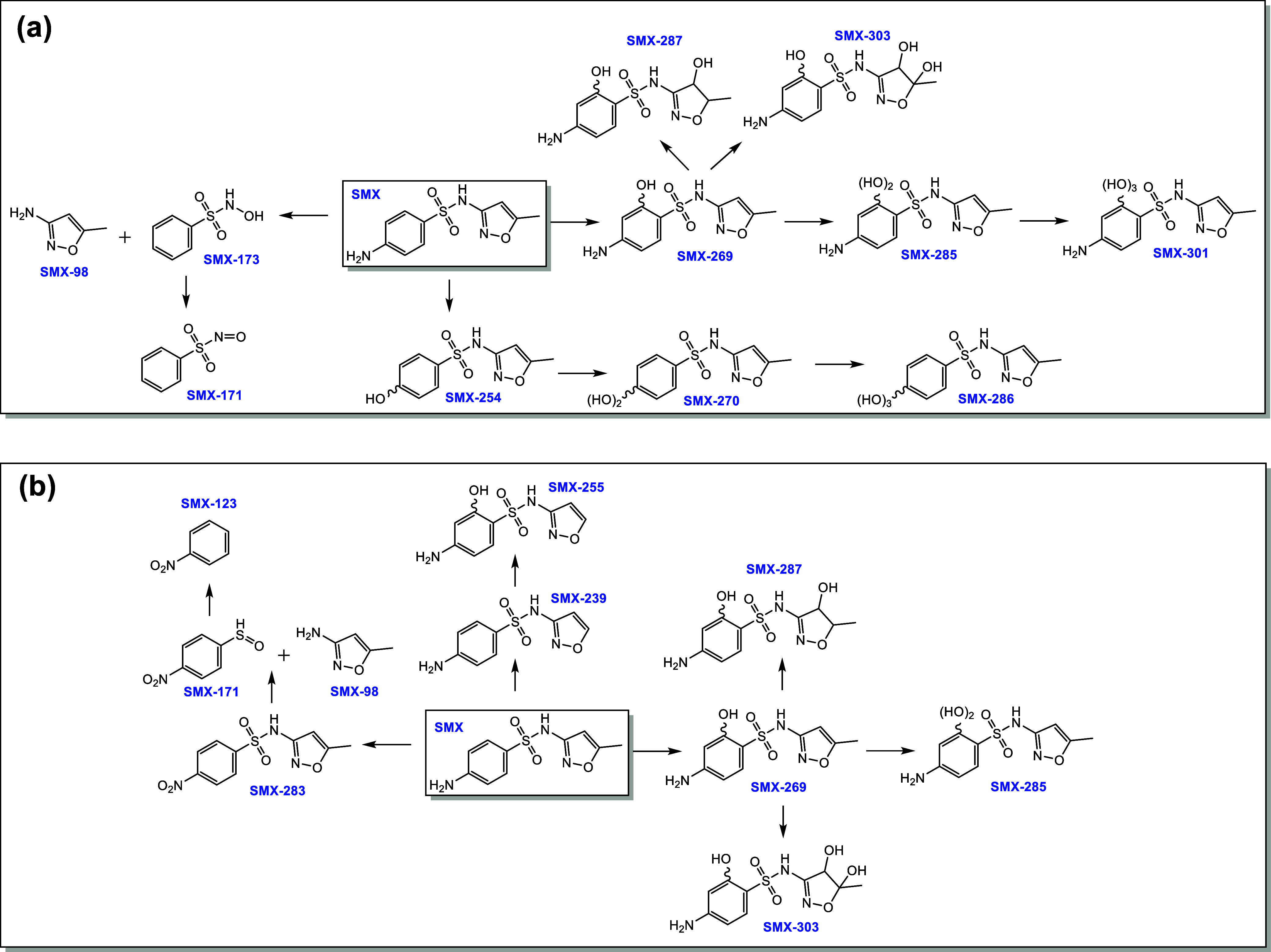
Possible transformation pathways for the advanced oxidation of sulfamethoxazole during (a) anodic oxidation and the Fenton process combined with anodic oxidation and (b) ozonation.

The SMX degradation pathway after ozonation treatment is shown in Figure 4b. For the electrochemical processes, hydroxylated intermediates were also detected during ozonation. However, hydroxylation here occurred via direct O_3_ cycloaddition to either the aryl or isoxazole ring, forming SMX-269/SMX-285 (aryl ring) and SMX-287/SMX-303 (isoxazole ring).^54,55^ The formation of SMX-283 involves the oxidation of the aromatic amino group (−NH_2_) to a nitro group (-NO_2_), a pathway previously reported.^56^ The inductive electron-withdrawing effect of the nitro moiety further weakens the S–N bond, which results in its cleavage after O_3_ attack, generating SMX-98 and SMX-171. These then undergo –SO loss to generate nitrobenzene (SMX-123).^57^ The demethylation of the isoxazole ring was also observed, resulting in the formation of SMX-239 (demethylated byproduct). This was followed by hydroxylation of the aromatic ring to generate SMX-255.^58^

### 5.2 Hydrochlorothiazide

The electrochemical treatment of HCTZ led to the generation of numerous transformation products in the simulated wastewater, as depicted in Figure 5a. The key transformation mechanisms include hydroxylation of the aromatic rings, heteroatomic ring opening, dehalogenation, and loss of the sulfonamide group. Chlorothiazide (HCTZ-295), a primary HCTZ intermediate,^59^ was tentatively identified and underwent the following changes: (i) dehalogenation to generate HCTZ-261; (ii) an increase in the O/C ratio through hydroxylation of the aromatic ring to form HCTZ-311; its subsequent dehalogenation led to HCTZ-277; and (iii) loss of the –NH_2_ group from the aromatic sulfonamide, leading to HCTZ-280; the subsequent elimination of the sulfonyl group and aromatic hydroxylation resulted in HCTZ-216 and HCTZ-232, respectively. Heteroatomic ring opening occurred via the cycloaddition of O_3_, resulting in the formation of HCTZ-285. The elimination of the sulfonamide moiety, followed by its hydroxylation, led to HCTZ-222. The sulfonate-derivate HCTZ-173 was formed by oxidation of the sulfonamide group and dehalogenation of HCTZ-222, whereas HCTZ-189 involved aromatic hydroxylation of the sulfonate-derivate.

**Figure 5:**
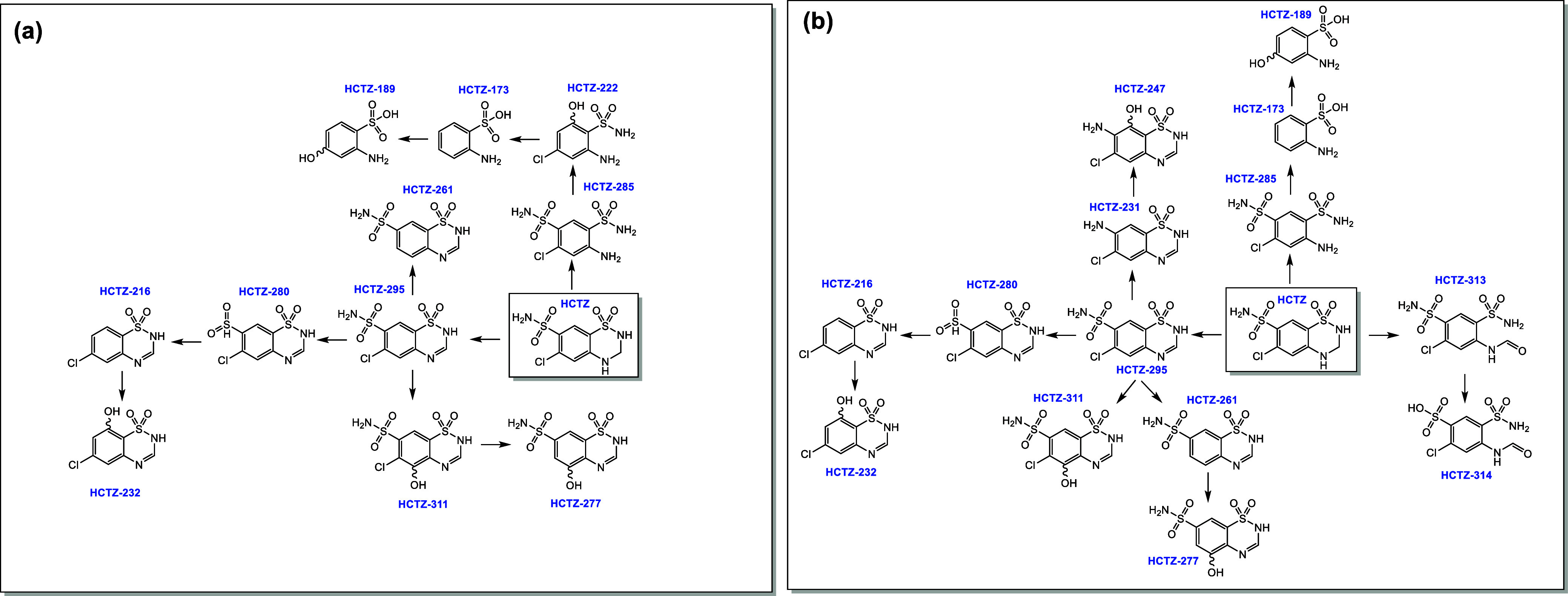
Possible transformation pathways for the advanced oxidation of hydrochlorothiazide during (a) anodic oxidation and Fenton process with anodic oxidation and (b) ozonation.

Several reaction intermediates were elucidated following HCTZ treatment with O_3_, as shown in Figure 5b. The amide-derivative HCTZ-313 was formed by heteroatomic ring opening after O_3_ cycloaddition, whereas HCTZ-314 corresponds to the sulfonate analog of HCTZ-313 (substitution of the –NH_2_ group by –OH).^60^ Similarly, HCTZ-285 was generated after heteroatomic ring opening, followed by the loss of formaldehyde;^61^ its subsequent dehalogenation and sulfonamide hydrolysis led to HCTZ-173, which was further hydroxylated to HCTZ-189. As in electrochemical oxidation, chlorothiazide (HCTZ-295) was also detected during ozonation.^62^ The hydroxylation and dehalogenation of this intermediate produced HCT-311 and HCTZ-261, respectively, and further hydroxylation of HCTZ-261 led to HCTZ-277. The loss of the –NH_2_ moiety from chlorothiazide formed HCTZ-280, and the elimination of the sulfonyl group produced HCTZ-219, which was further hydroxylated to HCTZ-232. The elimination of the –SO_2_ moiety from the sulfonamide^58^ of chlorothiazide resulted in HCTZ-231, and the hydroxylation of the aromatic ring led to HCTZ-247.

## 6 Energy Consumption and Costs

The energy consumption for the AO and EF + AO were calculated for the simulated wastewater as specific energy consumption (*E*_sp_), which is the energy consumed per gram of TOC removed and is expressed as the equation below

12 $$E_{sp}=\frac{V\times I\times t}{v\left({TOC}_{in}-{TOC}_{f}\right)}$$

where *E*_sp_ is the specific energy (kWh g^–1^ TOC), *V* is the voltage of the electrochemical cell (V), *I* is the applied current (A), *t* is the electrolysis time (h), *v* is the volume of the reacting mixture (L), TOC_in_ is the TOC at *t* = 0, and TOC_f_ is the TOC after the electrolysis time. Based on eq 12, the *E*_sp_ values for AO at 300 mA and EF + AO at 300 mA with the addition of 11.1 mg L^–1^ Fe^2+^ were calculated to be 0.29 kWh g^–1^ TOC and 0.12 kWh g^–1^ TOC, respectively. The nonhousehold electricity prices in Europe were averaged at 0.2008 € kWh^–1^ (Electricity price statistics, n.d.), implying electricity costs of 0.06 € g^–1^ TOC (AO) and 0.024 € g^–1^ TOC (EF + AO).

For ozonation, an ozone dose of 2 g O_3_ g^–1^ TOC was applied. The energy required to produce 1 kg O_3_ is approximately 10 kWh (value provided by the supplier of the ozone generation unit), implying that an energy of 0.02 kWh g^–1^ TOC is consumed in the ozonation process. Using 0.2008 € kWh^–1^, this corresponds to an electricity cost of approximately 0.004 € g^–1^ TOC. Notably, these costs correspond to the experimental conditions for which TOC removal plateaued at 23.76% in this water matrix. Although the cost of ozonation is low compared with that of AO or EF + AO, its achievable TOC removal under the tested conditions was limited. For higher mineralization targets, ozonation alone may not be sufficient and may require integration with downstream processes.

## 7 Conclusions

This study benchmarked three advanced oxidation routes (ozonation (O_3_), BDD-based anodic oxidation (AO), and the combined AO + electro-Fenton process (EF + AO)) for the removal of sulfamethoxazole (SMX) and hydrochlorothiazide (HCTZ) in a simulated matrix and a real municipal effluent. Under the investigated conditions, complete removal of both parent compounds was achieved by O_3_ and EF + AO (AO achieved an approximately 90% removal), with SMX consistently degrading faster than HCTZ. In addition to kinetics and mineralization, transformation products were identified and possible degradation pathways were proposed for each process, enabling a direct comparison of oxidation selectivity across technologies.

While ozonation provided the fastest abatement of the parent compounds at near-neutral pH, its mineralization was limited (24% TOC removal), consistent with prior studies reporting that ozonation often produces partially oxidized intermediates and may require post-treatment to enhance mineralization.^16^ In contrast, BDD-based AO and EF + AO achieved substantially higher mineralization (74% and 88%, respectively), in line with reports that electrochemical AOPs can drive deep oxidation via electrogenerated HO^•^.^19,25^

Matrix effects were most pronounced for ozonation: in the real effluent, TOC removal decreased by 54% relative to the simulated matrix, likely due to oxidant and radical scavenging by effluent organic matter and inorganic ions. AO and EF + AO were less sensitive (35% and 3% decrease, respectively), indicating that the combined EF + AO configuration can sustain mineralization even in complex matrices.

From an implementation perspective, each technology presents distinct trade-offs. Ozonation is a mature full-scale option and requires no pH adjustment. However, for matrices with high background organic carbon, ozone dose and achievable mineralization may be limiting. EF + AO delivered the highest mineralization and robustness, but requires pH adjustment to ∼3 and iron addition, which can influence downstream handling and operating cost. The comparative data set and transformation-product mapping reported here provide practical guidance for selecting and integrating AOP/eAOP technologies as tertiary treatment for pharmaceutical micropollutants. Future work should focus on process optimization and hybrid treatment trains (e.g., ozonation followed by biofiltration or eAOP polishing) to maximize both contaminant abatement and mineralization while minimizing energy and chemical inputs.
